# Supplementary material for: GLP-1/GIP Uptake, Indication, and Access Pathways Among US Adults in the Understanding America Study
Source: medRxiv. 2026 Sep 2:2026.08.28.26361368. Preprint. [Version 1] doi: 10.64898/2026.08.28.26361368 (PMC13684718; doi:10.64898/2026.08.28.26361368)
Supplement: Supplementary file 1 [file NIHPP2026.08.28.26361368V1-supplement-1.pdf]

medRxiv preprint doi: https://doi.org/10.64898/2026.08.28.26361368; this version posted September 2, 2026. The copyright holder for this preprint (which was not certified by peer review) is the author/funder, who has granted medRxiv a license to display the preprint in perpetuity.

It is made available under a CC-BY 4.0 International license .

Online Appendix

Title: GLP-1/GIP Uptake, Indication, and Access Pathways Among US Adults in the Understanding America Study, A Nationally Representative Cohort Study.

Ritika Chaturvedi PhD,[1*±] Tadeja Gracner PhD,[1,2*±] Francisco Perez-Arce, PhD[1], Sze-chuan Suen, PhD[3,4], Jing Jin, MS[3], Bart Orriens, PhD,[1] Rosalie Liccardo Pacula, PhD[4], Alison Sexton Ward, PhD[4], Rebecca Haile, BS[1], Arie Kapteyn, PhD[1]

1. Center for Economic and Social Research, University of Southern California, 635 Downey Way, Los Angeles, CA 90089

2. RAND Corporation, Santa Monica, 1776 Santa Monica, Los Angeles 90401

3. Viterbi School of Engineering, University of Southern California, Los Angeles, CA 90089

4. Schaeffer Center for Health Policy & Economics, University of Southern California, 635 Downey Way, Los Angeles, CA 90089.

*shared first authorship

±shared corresponding authorship: ritikac@usc.edu; (213) 740-7715; gracner@usc.edu; (510) 684-2716

Contents:

eFigure 1. *Schematic representation of individual UAS surveys used in analysis.*

eTable 1. *GLP-1/GIP –relevant individual data collected longitudinally in UAS (not exhaustive)*

eTable 2. *Weight-loss trials effects and pooled effects*

eTable 3. *Cardiovascular outcomes trials effects and pooled effects*

medRxiv preprint doi: https://doi.org/10.64898/2026.08.28.26361368; this version posted September 2, 2026. The copyright holder for this preprint (which was not certified by peer review) is the author/funder, who has granted medRxiv a license to display the preprint in perpetuity.
It is made available under a CC-BY 4.0 International license .

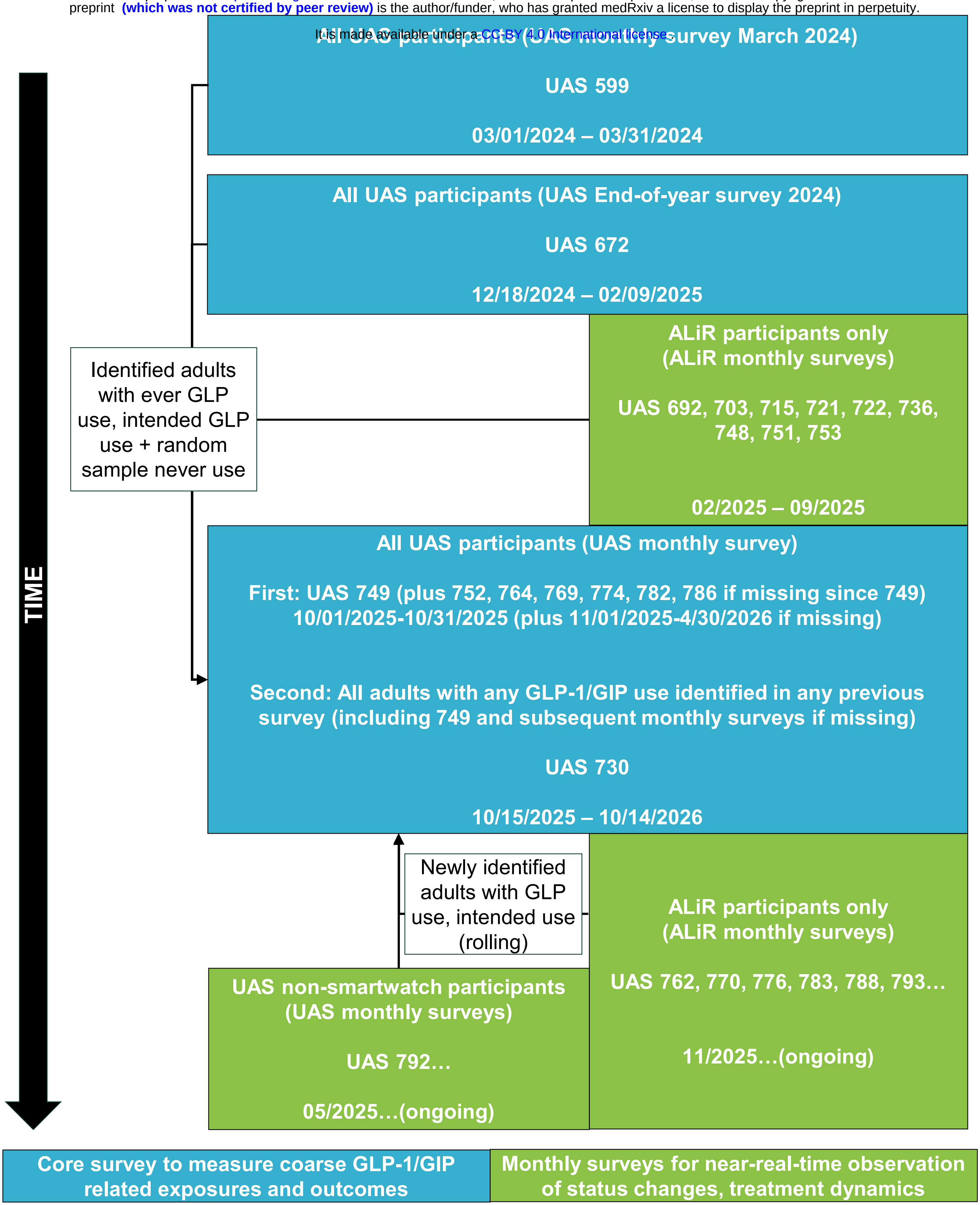

eFigure 1 *Schematic representation of individual UAS surveys used in analysis.*

medRxiv preprint doi: https://doi.org/10.64898/2026.08.28.26361368; this version posted September 2, 2026. The copyright holder for this preprint (which was not certified by peer review) is the author/funder, who has granted medRxiv a license to display the preprint in perpetuity.
It is made available under a CC-BY 4.0 International license .

	Electronic Surveys		Digital RWD	Linkages
	Repeated every 1-2 years	Repeated every 1-3 months	Continuous	Continuous
**GLP-1/GIP Treatment Experiences and Outcomes**				
Treatment Engagement	• *Uptake*<br>• *Likelihood of Future Use*<br>• *Reasons for Use*<br>• *Reasons for Discontinuation/Gaps*<br>• *Goals, Expected Outcomes*	• *Treatment Milestones (Indication, Initiation, Gaps, Discontinuation, Off-Label Behaviors, Agent, Administration, Dose Escalation)*<br>• *Relevant Dates for Treatment Milestones*		
Access, Insurance		• *Insurance Type and GLP-1/GIP Coverage*<br>• *GLP-1/GIP Availability, Accessibility*<br>• *GLP-1/GIP-Related Costs*<br>• *Barriers to Use*		
Healthcare Utilization	• *Prevention / Screening Use*<br>• *Healthcare History*<br>• *Healthcare Experience/Satisfaction*	• *Utilization, Service Type, Provider Type*<br>• *Healthcare Costs*		
Safety	• *Long Term (Muscle Loss, Frailty, Hormonal Symptoms, Eye Disorders, etc.)*	• *Short Term (e.g., Nausea and Gastrointestinal, Appetite, Nutrient Deficiencies, Disordered Eating, Fatigue, Sleep Disturbance, etc.)*		
**GLP-1/GIP Health Behaviors**				
Exercise	• *Self-reported Physical Activity*		• *Active / Sedentary Time*<br>• *Energy Expenditure*<br>• *Step Count*<br>• *Distance Covered*<br>• *Walking Speed*<br>• *Gait Irregularity*	
Diet		• *Diet*<br>• *Sugar Consumption*		
Sleep		• *Sleep Behaviors and Hygiene*	• *Time in Bed*<br>• *Sleep / Wake Onset*<br>• *Nap Number, Timing*	
Substance Use, Addiction	• *Substance Use Disorder*	• *Drugs Use, Type, Quantity*<br>• *Alcohol Use, Quantity*<br>• *Nicotine Use, Modality, Quantity*<br>• *Gambling*<br>• *Cravings*<br>• *Social Media / Screen Time*		
**GLP-1/GIP Related Health Outcomes**				
Health Status	• *Self-Reported Health*	• *General Health*<br>• *Well-being. Quality of Life*	• *Basal Metabolic Rate*<br>• *Heart rate variability*<br>• *VO_2 max*<br>• *SpO_2*<br>• *Breathing Rate*<br>• *Skin Temperature*	
Physical Health	• *Chronic Condition Diagnoses*<br>• *Cardiovascular Outcomes (MACE: heart attack, stroke, heart failure, arrhythmia, etc.)*<br>• *Medications*<br>• *Health History*	• *Activities of Daily Living*<br>• *Weight, Body Mass Index*<br>• *Blood Sugar*<br>• *Acute Symptoms (e.g., Respiratory, Gastrointestinal, Headache, Malaise, etc.)*<br>• *Women's Health, Pregnancy*	• *Resting / Active Heart Rate*<br>• *Circadian Rhythm*<br>• *Interstitial Glucose (upcoming)*<br>• *Glycemic Variability (upcoming)*	• *CMS Diagnoses and Claims*
Sleep Health	• *Sleep Disorders (e.g., Sleep Apnea)*	• *Sleep Disturbance, Satisfaction*	• *Sleep Duration*<br>• *Sleep Quality*<br>• *Sleep Stages*<br>• *Chronotyping*	
Mental Health	• *Mental Illness Diagnoses*	• *Depression*<br>• *Anxiety*<br>• *Pain*<br>• *Stress*		
Cognitive Health	• *Standardized Cognitive Tests (Verbal, Reasoning, Executive Function, Intelligence)*<br>• *Cognitive Impairment Status*	• *Standardized Cognitive Tests (Working / Episodic Memory, Perceptual Speed)*	• *Functional Ability*	• *Cognitive Impairment, Dementia Diagnoses*
**Environmental Factors**				
Location, Neighborhood	• *Built Environment, Neighborhood Features*<br>• *Transportation*<br>• *Walkability*	• *Zip Code / Census Region*<br>• *Urbanicity*<br>• *Perceived Safety*<br>• *Police Presence*	• *Wearable Air Quality*	• *Air / Water Quality, Weather*<br>• *Crime*<br>• *National Walkability Index (upcoming), USDA Food Environment (upcoming)*
Housing, Food Security	• *Housing Type / Characteristics / History*<br>• *Housing / Utility Stability*	• *Food Security*		
Social Network	• *Social Network*<br>• *Culture / Religiosity / Politics*<br>• *Racism, Discrimination*	• *Household*<br>• *Marital Status / Family Structure*<br>• *Social Support, Loneliness*		
**Economic Factors**				
Education, Competencies	• *Education*<br>• *Financial Literacy*<br>• *Digital Literacy*<br>• *Health Literacy*		• *Digital Engagement*<br>• *Digital Proficiency*	
Work and Finance	• *Wealth / Debt*<br>• *Financial Wellbeing*	• *Labor Force Status / Occupation*<br>• *Productivity, Absenteeism*<br>• *Income*		
**Intrinsic Factors**				
Demographics, Personality	• *Personality*<br>• *Decision Making*<br>• *Risk Tolerance*<br>• *Mood / Affect*<br>• *Life Satisfaction*<br>• *Self-Esteem*	• *Age*<br>• *Race / Ethnicity*<br>• *Sex / Gender Identity*<br>• *Sexual Orientation*<br>• *Primary Language*<br>• *Health Motivations*		• *Genomics and Polygenic Risk Scores*

Notes: *Core Understanding America Study survey measures, repeated quarterly for sociodemographics and at least biennially for other variables, are available from 2014.*

eTable 1 *GLP-1/GIP –relevant individual data collected longitudinally in UAS (not exhaustive)*

medRxiv preprint doi: https://doi.org/10.64898/2026.08.28.26361368; this version posted September 2, 2026. The copyright holder for this preprint (which was not certified by peer review) is the author/funder, who has granted medRxiv a license to display the preprint in perpetuity.

It is made available under a CC-BY 4.0 International license .

**Trial**	**RCT-STEP 1**	**RCT-SURMOUNT-1**	**RCT-SURMOUNT-5**	**RCT-SURMOUNT-6**	**Pooled**
Semaglutide	X			X	44.50%
Tirzepatide		X	X		55.50%
female	74.10%	67.50%	64.70%	64.60%	69.60%
age	46± 13	44.9± 12.5	45.0±12.9	44.4±12.7	45.3 ± 12.7
BMI	37.9± 6.7	38± 6.81	39.4±7.4	39.4±7.7	38.2 ± 6.9
weight(kg)	105.3±21.9	104.8 ± 22.12	112.7±24.8	113.4±26.3	106.2 ± 22.7
weight(lb)	232.1 ± 48.3	231 ±48.8	249.1 ± 56.4	250.0 ± 58.0	234.1 ± 50.3
diabetes	0%	0%	0%	0%	0%
hypertension	36.10%	32.30%	41.70%	37.50%	34.80%
cardiovascular disease	2.5%	3.1%	1.3%	1.6%	2.6
**Total N**	**1961**	**2539**	**374**	**376**	**5250**

eTable 2. *Weight-loss trials effects and pooled effects*

medRxiv preprint doi: https://doi.org/10.64898/2026.08.28.26361368; this version posted September 2, 2026. The copyright holder for this preprint (which was not certified by peer review) is the author/funder, who has granted medRxiv a license to display the preprint in perpetuity.

It is made available under a CC-BY 4.0 International license .

Trial	ELIXA	LEADER	SUSTAIN-2	SUSTAIN-2	SUSTAIN-2	SUSTAIN-3	SUSTAIN-3	SUSTAIN-6	EXSCEL	Harmony	REWIND	PIONEER 6	PIONEER 3	PIONEER 3	PIONEER 3	PIONEER 3	PIONEER 4	PIONEER 4	AMPLITUDE	SURPASS-1	SURPASS 2	SURPASS 2	SURPASS 2	SURPASS 2	Pooled
Lixisenatide	X																								8%
Liraglutide		X																X							12%
Semaglutide			X	X		X		X				X	X 3 mg/d	X 7 mg/d	X 14 mg/d		X							X	20%
Sitagliptin																X									2%
Exenatide							X		X																19%
Albiglutide										X															12%
Dulaglutide											X														12%
Efpeglenatide																			X						10%
Tirzepatide																				X	X	X	X		5%
Female	30.%	35%	49%	50%	49%	45%	43%	39%	38%	30%	46%	31%	45%	47%	46%	51%	48%	48%	33%	28%	56%	49%	54.5	52%	38%
Age	59.9+-9.7	64.2+-7.2	54.8+-10.2	56.0+-9.4	54.6+-10.4	56.4+-10.3	56.7+- 11.1	64.6 +- 7.4	62(56, 68)	64.1 +- 8.7	66.2 +- 6.5	66 +- 7	58 +- 10	58 +- 10	57 +- 10	58 +- 10	56+-10	56+-10	64.5+-8.2	54.10+- 11.88	56.3±10.0	57.2±10.5	55.9±10.4	56.9±10.8	62.7 ± 9.2
BMI	30.1+-5.6	32.5 +- 6.3	32.4+-6.2	32.5+-6.6	32.5+-5.8	NR	NR	32.8+-6.2	31.8	32.3+-5.9	32.3 +- 5.7	32.3 +- 6.6	32.6+-6.7	32.6+-6.4	32.3+-6.3	32.5+-6.2	32.5 +- 5.9	33.4 +- 6.7	32.7±6.2	NR	33.8±6.85	34.3±6.60	34.5±7.11	34.2±7.15	32.3 ± 6.2
weight(kg)	84.6+-19.2	91.9 +- 21.2	89.9+-20.4	89.2+-20.7	89.3+-19.7	96.21 +- 22.5	95.37+- 20.46	92.1 kg +- 20.6	NR	NR	NR	91.0±21.4	91.6+-22	91.3+-20.8	91.2+-21.7	90.9+-21	92.9+- 20.6	95.5+-21.9	NR	NR	92.5±21.76	94.8±22.71	93.8±21.83	93.7±21.12	90.9 ± 21.1
weight(lb)	186.5 ± 42.3	202.6+-46.7	198.20 ± 44.97	196.65 ± 45.64	196.87 ± 43.43	212.11 ± 49.60 lb	210.25 ± 45.11	203.04 ± 45.42	NR	NR	NR	200.62 ± 47.18	201.94 ± 48.50	201.28 ± 45.86	201.06 ± 47.84	200.40 ± 46.30	204.81 ± 45.42	210.54 ± 48.28	NR	NR	206.57 ± 48.19	208.99 ± 50.07	206.79 ± 48.13	206.57 ± 48.19	200.4 ± 46.5
diabetes	100%	100%	100%	100%	100%	100%	100%	100%	100%	100%	100%	100%	100%	100%	100%	100%	100%	100%	100%	100%	100%	100%	100%	100%	100%
hypertension	75.60%	92.7%***	NR	NR	NR	NR	NR	92.80%	NR	86%	93%	23.9% ^	NR	NR	NR	NR	NR	NR	91.30%	NR	NR	NR	NR	NR	85.20%
cardiovascular disease	100%	82.1%^^^	NR	NR	NR	NR	NR	60.5%****	83.00%	100%	31.50%	84.7%^^	NR	NR	NR	NR	NR	NR	89.6	NR	NR	NR	NR	NR	78%
**Total N**	**3034**	**4,668**	**409**	**409**	**407**	**404**	**405**	**3,297**	**7356**	**4731**	**4949**	**1591**	**466**	**465**	**465**	**467**	**285**	**284**	**4076**	**478**	**470**	**469**	**470**	**469**	**40524**

eTable 3. *Cardiovascular outcomes trials effects and pooled effects*
